# High TRIM44 expression as a valuable biomarker for diagnosis and prognosis in cervical cancer

**DOI:** 10.1042/BSR20181639

**Published:** 2019-03-06

**Authors:** Shuang Liu, Fanling Meng, Jing Ding, Hongying Ji, Mu Lin, Jiaqi Zhu, Rong Ma

**Affiliations:** Department of Gynecology, Harbin Medical University Cancer Hospital, Harbin, China

**Keywords:** Cervical cancer, Metastasis, Prognosis, TRIM44

## Abstract

Tripartite motif containing 44 (TRIM44) has been reported to be up-regulated in multiple aggressive malignant tumors. However, its expression status and clinical significance in cervical cancer remain unknown. The purpose of this study was to investigate the clinical significance of TRIM44 expression and the prognosis in patients with cervical cancer (CC). Fresh frozen tissues from 5 samples of CC and 4 normal cervical tissues were analyzed for TRIM44 expression using RT- PCR and Western blot analysis. 122 paraffin-embedded surgical specimens from patients with CC were collected for an immunohistochemistry. TRIM44 expression was found to be significantly up-regulated in cervical cancer specimens compared with adjacent normal tissues (*P*<0.001). Statistical analysis showed that TRIM44 expression was significantly correlated with the International Federation of Gynecology and Obstetrics (FIGO) stage, histological grade and lymph node metastasis, but not with age, histological type, and tumor size. Kaplan–Meier survival analysis suggested that high TRIM44 expression was associated with poor prognosis. Patients highly expressing TRIM44 have significantly shorter overall survival (OS) (*P*=0.006) and disease-free survival (DFS) (*P*=0.002). Furthermore, multivariate Cox analysis showed TRIM44 was an independent risk factor for poor prognosis. Our study demonstrated that TRIM44 expression contributes to the progression of cervical cancer, and could be used as a marker of clinical diagnosis and prognosis of patients with cervical cancer.

## Introduction

Cervical cancer continues to be the third most common malignancy in women worldwide [1], with an estimated global incidence of over 500000 new cases every year and approximately 265700 women deaths from this disease per year [2].

Although vaccination and screening for cervical cancer are helpful for prevention, early detection and standard treatments, such as radiotherapy, chemotherapy, and surgery, can lead to remission; however, clinical prognosis still varies significantly and is not easy to predict [3–7]. Therefore, it is significant to explore the molecular pathogenesis of cervical cancer and identify prognostic markers to improve therapeutic strategies, and thus tumor biological behavior would be prognosticated reliably, effective treatment would be prescribed, patient care would be improved considerably.

To date, several studies have reported that Tripartite motif containing 44 (TRIM44), a crucial member of the tripartite motif-containing protein (TRIM) family [8], has been found to participate in diverse pathological conditions, such as cancer, developmental disorders, neurodegenerative diseases, and viral infections [9]. It has been demonstrated that Trim44 is involved in the development and progression of several malignant tumors [8–16]. TRIM44 overexpression has been reported in a diversity of tumors, including prostate cancer [8], gastric cancer [10], hepatocellular carcinoma (HCC) [11], intrahepatic cholangiocarcinoma [12], testicular germ cell tumor [13], lung cancer [9,14], and esophageal carcinoma [16,17]. These previous studies have revealed that TRIM44 is involved in the migration and invasion of malignant tumors, which indicated that TRIM44 may be useful as a clinically relevant prognostic biomarker and a new therapeutic target in the future. All these studies suggest that TRIM44 plays a considerable role in tumor development. However, the potential involvement of TRIM44 in cervical cancer and the related mechanisms have not been fully explored.

In the present study, we investigated the expression of TRIM44 in cervical cancer and assessed the role of TRIM44 in the progression of cervical cancer. The results of the present study suggest that TRIM44 expression was significantly up-regulated in cervical cancer specimens compared with adjacent normal tissues and was significantly associated with poor prognosis. Our study demonstrated that TRIM44 expression contributes to the progression of cervical cancer, and could be used as a marker of clinical diagnosis and prognosis of patients with cervical cancer.

## Materials and methods

### Patient population

For the present study, 122 specimens archived on formalin-fixed, paraffin-embedded tissue blocks were obtained between 2010 and 2013 from the Harbin Medical University Cancer Hospital. One hundred and nine cases were squamous cell carcinoma. The other 13 cases were adenocarcinoma. All cases were diagnosed according to the criteria of the International Federation of Gynecology and Obstetrics (FIGO). No patient received neoadjuvant chemotherapy, radiotherapy, or other treatment prior to surgery. All patients underwent a radical hysterectomy and pelvic lymphadenectomy. The present study was approved and supervised by the Ethical Committee of Harbin Medical University Cancer Hospital. Normal cervical tissues in the present study were selected from women undergoing hysterectomy for the benign uterine disease at the Department of Gynecology of the Harbin Medical University Cancer Hospital. Detailed clinicopathological features were summarized in Table 1.

**Table 1:** Association analyses between the expression levels of TRIM44 and the clinicopathological characteristics of CC

Variables	Patients, *n*	TRIM44 expression		*P*^1^
		Low	High	
All cases				
Age (years)				
≤55	57	15	42	*P*=0.127
>55	65	26	39	
FIGO stage				
	53	28	25	*P*<0.001
Ⅱ	69	13	56	
Histological grade				
G1	39	22	17	*P*<0.001
G2/G3	83	19	64	
Histological type				
SCC	109	34	75	*P*=0.125
Adenocarcinoma	13	7	6	
Tumor size				
≤4 cm	93	35	58	*P*=0.116
>4 cm	29	6	23	
Lymph node metastasis				
No	104	39	65	*P*=0.032
Yes	18	2	16	

Abbreviations: CC, cervical cancer; G1, well differentiated; G2, moderately differentiated; G3, poorly differentiated; SCC, squamous cell carcinoma. ^1^Chi-square test.

Patient consent and approval from the Medical Ethical Committee of The Harbin Medical University Cancer Hospital were obtained for the purpose of research. The research has been carried out in accordance with the World Medical Association Declaration of Helsinki, and that all subjects provided written informed consent.

### Western blot analysis

Tissues were lysed, protease inhibitors added to the lysates and centrifuged at 12000 rpm at 4°C. Proteins were extracted from tissue samples by using RIPA buffer (Beyotime). Proteins were separated by SDS/PAGE, transferred on to PVDF membranes (Sigma, St. Louis, MO, U.S.A.) and detected with primary antibodies against TRIM44 (1:300, Novus Biologicals, LLC, U.S.A.) or β-actin (1:1000, Santa Cruz Biotechnology, Santa Cruz, CA, U.S.A.). The membranes were incubated at 4°C overnight. The ECL chemiluminescent kit (Millipore, MA, U.S.A.) was applied to visualize protein bands.

### Immunohistochemical staining and assessment

Immunohistochemistry was performed using an anti-TRIM44 antibody following standard method. In brief, paraffin-embedded samples were sectioned into 5-μm-thick slices and stained with Hematoxylin. After deparaffinization and rehydration, endogenous peroxidases were quenched by incubating the sections for 10 min in 3% H_2_O_2_. TRIM44 antigen retrieval was used by heating the sections in a stainless autoclave. After washing in PBS, the sections were incubated at 4°C overnight with an anti-TRIM44 (1:100) antibody in a humid chamber. In addition, the sections were washed in PBS, incubated with secondary antibodies and DAB, and then counterstained with Hematoxylin. Slides were counterstained with Hematoxylin for 2 min.

For assessment of TRIM44 expression, the immunohistochemistry scoring procedure was carried out independently by two pathologists blinded to the clinical data and the staining intensity and relative percentage of immunostained cells were evaluated for each case. The intensity of immunostaining was documented as follows: 0 (negative), 1 (weak), 2 (moderate), and 3 (strong) in at least five different high-power fields. The percentage of immunostained cells was scored as ‘0’ for < 10%, ‘1’ for 10–33%, ‘2’ for 34–66%, and ‘3’ for 67–100%. TRIM44 protein expression levels were classified by the total combined score of positive-staining tumor cell percentage and staining intensity. By this means, we divided the samples into two groups: low expression (<3) and high expression (≥3).

The scoring procedure was performed at least twice by two independent pathologists who were experienced in pathological research and had no knowledge of the clinicopathological characteristics.

### Real-time PCR

The expression of *TRIM44* mRNA was quantitated by real-time RT-PCR. Briefly, total RNA was extracted from cervical cancer tissues and normal cervical tissues (*n*=9) using TRIzol reagent (Invitrogen Life Technologies, Carlsbad, CA, U.S.A.) according to the manufacturer’s protocol. The Superscript III Platinum Kit (Invitrogen) was used to reverse transcribe this total RNA into cDNA. Real-time PCR was performed with SYBR Green Master Mix (TaKaRa, Kyoto, Japan) using the following primers against TRIM44: Forward, 5′- GGCTTGATTTGAGTACCTATT-3′; Reverse, 5′-AGTCCACCTGAGTCTTTGC-3′. β-actin served as an internal reference; its expression was analyzed using the following primers: Forward, 5′-CGGGAAATCGTGCGTGAC-3′; Reverse, 5′-GTCAGGCAGCTCGTAGCTCTT-3′. Relative quantitation of *TRIM44* mRNA was conducted using the 2^−ΔΔ*C*^_T_ method, followed by normalization using the internal control.

### Statistical analysis

Differences between categorical variables were determined using Pearson’s chi-square test or Fisher’s exact test as indicated. Differences in overall survival (OS) and disease-free survival (DFS) between patient groups were assessed using Kaplan–Meier curves and log-rank test. The Cox proportional hazards model was used for multivariate analysis of prognostic predictors for OS and DFS. *P*-values <0.05 were considered statistically significant and all tests were two-sided. All statistical analyses were performed using SPSS 21.0 for Windows (SPSS, Chicago, IL, U.S.A.).

## Results

### Clinicopathological features of patients with cervical cancer

A total of 122 cervical cancer specimens archived on formalin-fixed paraffin-embedded tissue blocks were included in the present study. The clinicopathological features of patients enrolled in the present study are summarized in Table 1. Amongst them, 39 (31.97%) patients were grade 1 (G1) and 83 patients (68.03%) were grade 2 and grade 3 (G2/G3). According to the FIGO staging criteria, 53 (43.44%) patients were stage I and 69 (56.56%) patients were stage II. There were 18 (14.75%) patients with lymph node metastasis at the time of diagnosis; 122 patients were further grouped into two histological types including 109 cases with squamous cell carcinoma and 13 cases with adenocarcinoma. There were 23 deaths during a median follow-up period of 65 months (range: 2–85 months). The 3- and 5-year OS and DFS were 93.4, 81.1% and 86.8, 76.8%, respectively.

### Expression pattern of TRIM44 in cervical cancer

**Figure 1:**
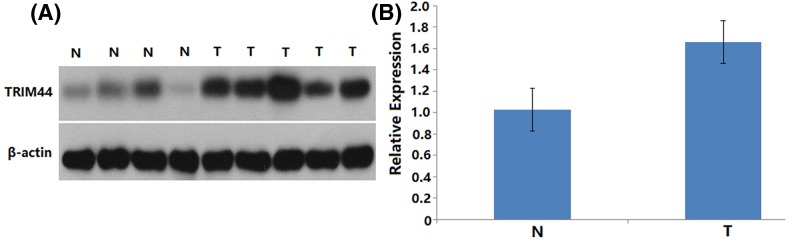
TRIM44 protein expression in normal and cervical cancer tissues (**A**) Protein samples obtained from frozen normal cervical tissues (N) and cervical cancer tissues (T) were analyzed by Western blot analysis. Levels of β-actin were used as an internal control. (**B**) Histogram of pooled data from N (*n*=4) and cervical cancer tissues (*n*=5). TRIM44 expression was elevated in cervical cancer tissues compared with adjacent normal tissues. The data are presented as mean ± S.D.

**Figure 2:**
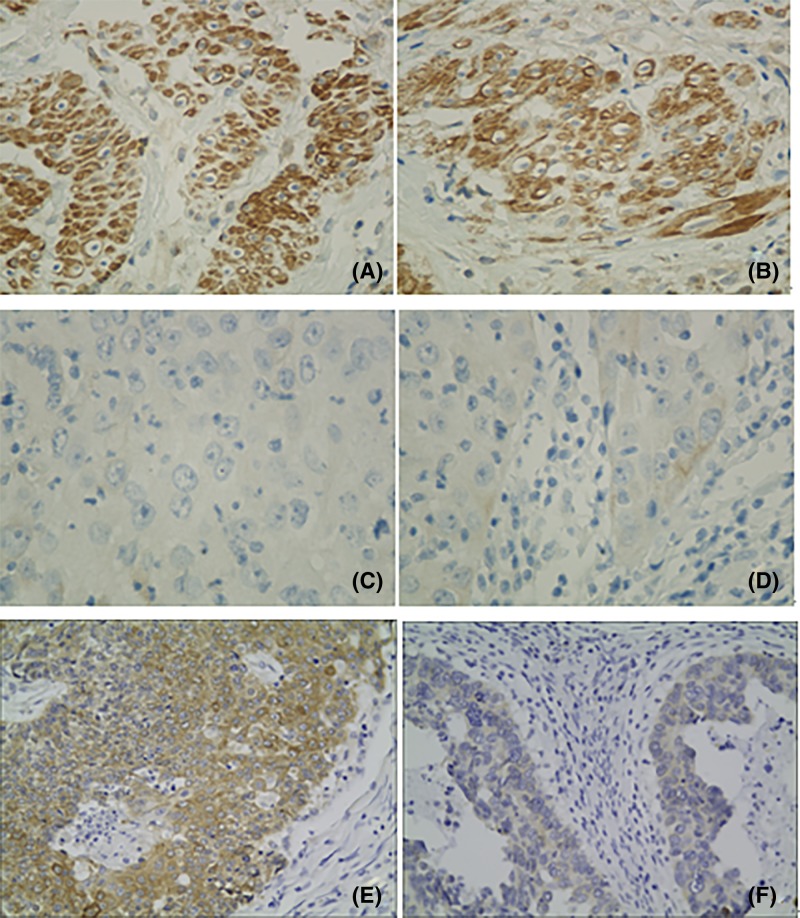
Immunohistochemical staining of TRIM44 in cervical cancer (**A**) High expression of TRIM44 in a high-grade cervical cancer. (**B**) High expression of TRIM44 in a low-grade cervical cancer. (**C**) Low expression of TRIM44 in a high-grade cervical cancer. (**D**) Low expression of TRIM44 in a low-grade cervical cancer. (**E**) High expression of TRIM44 in a low-grade cervical adenocarcinoma. (**F**) Low expression of TRIM44 in a low-grade cervical adenocarcinoma.

To investigate the expression pattern of TRIM44 in CC, the expression levels of TRIM44 in 5 CC tissues and 4 normal tissues were detected using Western blotting and RT-PCR. As shown in Figure 1, significant statistical differences in TRIM44 expression were observed between cervical cancer tissues and their adjacent normal tissues. The mRNA and protein level of TRIM44 was significantly higher in cervical cancer tissues compared with adjacent normal tissues (*P*<0.001, Figure 1). The immunohistochemistry analysis showed that TRIM44 expression was localized in the cytoplasm of cervical cancer cells (Figure 2). Of the 122 cervical cancer cases, 41 (33.6%) and 81 (66.4%) showed low and high TRIM44 expression, respectively.

**Figure 3:**
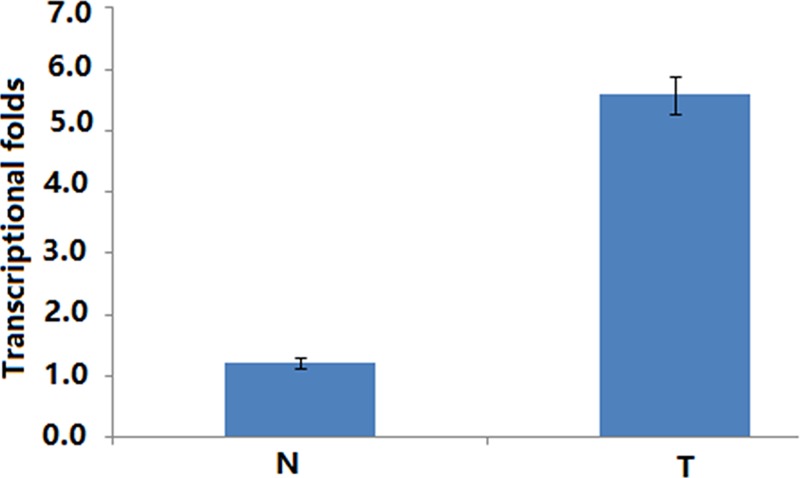
Histogram of *TRIM44* mRNA expression in normal cervical tissues and cervical cancer tissues Levels of β-actin were used as an internal control, and the *TRIM44* mRNA expression was calculated by 2^−ΔΔ*C*^_t_ method. *TRIM44* mRNA expression was elevated in CCs compared with normal cervical tissues. The data are presented as mean ± S.D. (*P*<0.05). Abbreviation: CC, cervical cancer.

RT-PCR result showed that the mean expression level of *TRIM44* mRNA was significantly higher in cervical cancer tissues than in normal cervical tissues (*P*<0.05, Figure 3).

### Expression of TRIM44 correlates with clinicopathological features and patients’ prognosis

According to TRIM44 protein expression levels expression, 122 patients were divided into two groups: low expression group (*n*=41) and high expression group (*n*=81). We first analyzed the relationship between TRIM44 expression and clinicopathological features. Statistical analysis showed that TRIM44 expression was significantly correlated with FIGO stage (*P*<0.001), histological grade (*P*<0.001), and lymph node metastasis (*P*=0.032), but not with age, histological type, and tumor size (Table 1). The high expression level of TRIM44 was correlated with a higher histological grade and FIGO stage compared with the patient group with low TRIM44 expression. The TRIM44 expression level was statistically higher in patients with lymph node metastasis than in those without lymph node metastasis.

**Figure 4:**
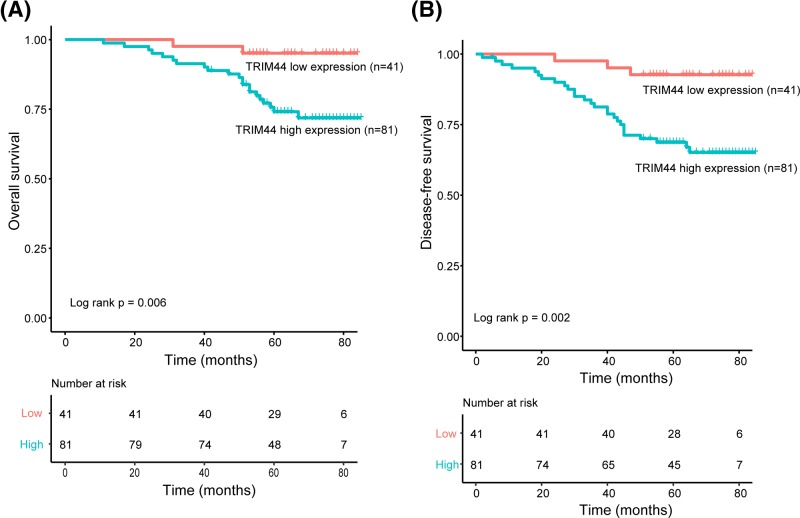
Expression of TRIM44 correlates with and patients’ prognosis (**A**) Kaplan–Meier curves for OS in patients with cervical cancer according to TRIM44 expression. (**B**) Kaplan–Meier curves for DFS in patients with cervical cancer according to TRIM44 expression.

We next analyzed the relationship between TRIM44 expression and patients’ prognosis. Results of univariate Cox analysis suggested that there were significant associations between TRIM44 expression and patients’ prognosis (*P*=0.006 for OS and *P*=0.002 for DFS). Our results showed that patients with cervical adenocarcinoma exhibited significantly shorter OS (*P*=0.004) and DFS (*P*=0.001) compared with patients with cervical squamous cell carcinoma (Figure 3 and Table 2). In addition, we also conducted a Kaplan–Meier analysis and found that there was a significant difference in OS and DFS between the two groups of patients stratified by TRIM44 expression level. Patients with high TRIM44 expression exhibited significantly shorter OS (*P*=0.006) and DFS (*P*=0.002) compared with patients with low TRIM44 expression (Figures 3 and 4). The 3-year OS rate and DFS rate of the high expression group were 91.4 and 81.3%, respectively, whereas the corresponding rates in the low expression group were 97.6 and 97.6%, respectively. The 5-year OS rate and DFS rate of the high expression group were 74.1 and 68.7%, respectively, whereas the corresponding rates in the low expression group were 95.1 and 92.7%, respectively.

**Table 2:** Univariate survival analysis of OS and DFS in 122 patients with CC

Variables	*n*	OS		*P*^1^	DFS		*P*^a^
		Mean ± S.E.M. (month)	95% CI		Mean ± S.E.M. (month)	95% CI	
Age (years)							
≤55	57	77 ± 2	72–81	*P*=0.969	73 ± 3	67–79	*P*=0.622
>55	65	75 ± 2	71–79		70 ± 3	64–76	
FIGO stage							
	53	79 ± 2	75–83	*P*=0.084	76 ± 3	71–81	*P*=0.153
Ⅱ	69	73 ± 2	69–78		68 ± 3	62–74	
Histological grade							
G1	39	78 ± 2	75–82	*P*=0.233	76 ± 3	70–81	*P*=0.103
G2/G3	83	75 ± 2	71–79		70 ± 3	64–75	
Histological type							
SCC	109	78 ± 2	75–81	*P*=0.004	74 ± 2	70–79	*P*=0.001
Adenocarcinoma	13	65 ± 6	54–76		54 ± 7	40–67	
Tumor size							
≤4cm	93	79 ± 2	76–82	*P*=0.002	76 ± 2	72–80	*P*<0.001
>4cm	29	66 ± 3	59–72		56 ± 5	46–65	
Lymph node metastasis							
No	104	79 ± 2	76–82	*P*<0.001	75 ± 2	71–79	*P*<0.001
Yes	18	60 ± 5	51–69		51 ± 6	39–63	
TRIM44 expression							
Low expression	41	82 ± 1	79–85	*P*=0.006	81 ± 2	77–84	*P*=0.002
High expression	81	74 ± 2	69–78		67 ± 3	62–73	

Abbreviations: CC, cervical cancer; CI, confidence interval; G1, well differentiated; G2, moderately differentiated; G3, poorly differentiated. ^1^Log-rank test.

### Overexpression of TRIM44 is an unfavorable prognostic factor independent of other clinicopathological features

University Cox analysis also suggested that histological type, tumor size, and lymph node metastasis were correlated with patients’ OS and DFS except for TRIM44 expression (Table 2). Therefore, we conducted a multivariate analysis using the Cox proportional hazards model to examine whether the prognostic value of TRIM44 expression is independent of other clinicopathological features. As shown in Table 3, the results from multivariate Cox analysis showed that TRIM44 expression (HR = 5.921, 95% confidence interval (CI) = 1.327–26.432, *P*=0.02 for OS; HR = 6.552, 95% CI = 1.881–22.82, *P*=0.003 for DFS), histological type (HR = 3.891, 95% CI = 1.393–10.865, *P*=0.01 for OS; HR = 5.412, 95% CI = 2.223–13.173, *P*<0.001 for DFS), and lymph node metastasis (HR = 3.108, 95% CI = 1.294–7.464, *P*=0.011 for OS; HR = 2.626, 95% CI = 1.187–5.807, *P*=0.017 for DFS) (Table 3). These results thus indicated that the prognostic value of TRIM44 expression as a molecular biomarker is independent of other clinicopathological factors.

**Table 3:** Multivariate survival analysis of OS and DFS in patients with CC

Variables	OS			DFS		
	Exp (B)	95% CI	*P*^1^	Exp (B)	95% CI	*P*^1^
Histological type	3.891	1.393–10.865	*P*=0.010	5.412	2.223–13.173	*P*<0.001
TRIM44 expression	5.921	1.327–26.432	*P*=0.020	6.552	1.881–22.820	*P*=0.003
Tumor size	2.164	0.900–5.204	*P*=0.085	2.409	1.138–5.101	*P*=0.022
Lymph node metastasis	3.108	1.294–7.464	*P*=0.011	2.626	1.187–5.807	*P*=0.017

Abbreviation: CC, cervical cancer. ^1^Cox regression test.

## Discussion

As for TRIM44, our study is the first report to assess the TRIM44 expression as well as its association with clinical pathological features in cervical cancer. We found that TRIM44 is overexpressed in cervical cancer and is closely related to tumor progression and unfavorable outcome.

In the present study, we found that the expression of TRIM44 was significantly higher in tumor tissues than in normal cervical tissues by both Western blotting and RT-PCR analysis. In addition, higher TRIM44 expression was related to CC featuring lower tumor differentiation and an advanced stage. The TRIM44 expression level was statistically higher in patients with lymph node metastasis than in those without lymph node metastasis. In general, higher expression of TRIM44 indicated a clinically more malignant cervical cancer. Importantly, univariate survival analysis revealed that CC patients with high TRIM44 expression were more likely to have shorter PFS and OS than those with low TRIM44 expression. Therefore, TRIM44 could be an oncogene for cervical cancer development and patient prognosis. Our results were quite consistent with those of previous studies [9–17]. Kashimoto et al. [10] reported that TRIM44 was overexpressed in gastric carcinoma tissues. Enhanced expression of TRIM44 was associated with an advanced type of macroscopic appearance, lymphatic invasion, and higher recurrence rate, and poor prognosis of patients with gastric cancer. In addition, knockdown of TRIM44 inhibited the proliferation, migration, and invasion of tumor cells.

Recent studies have demonstrated a multifaceted role of TRIM44 in cancer progression [14,18–20]. For example, a recent investigation indicates that high expression of TRIM44 promotes cell proliferation via accelerating the G_1_/S phase transition and enhance the invasive and migratory capacity of HCC cells. Meanwhile, TRIM44 is involved in resistance of HCC cells to doxorubicin by way of accelerating NF-κB activation [11]. Consistent with the previous report, TRIM44 facilitates the migration and invasion of human lung cancer cells via the NF–κB signaling pathway [14] and in another preclinical study, while TRIM44 promotes proliferation and metastasis in non–small cell lung cancer via mTOR signaling pathway [9]. Overexpression of TRIM44 contributes to malignant outcome in gastric carcinoma, and forebodes a worse OS than those with non-expressing tumors [10]. In agreement with previous results, TRIM44 promotes cell proliferation and migration, and inhibits apoptosis in testicular germ cell tumor [13]. It has been reported that knockdown of TRIM44 inhibits the proliferation and invasion in prostate cancer cells. These results demonstrated that overexpression of TRIM44 in carcinoma significantly facilitates the invasion and migration. As stated above, these data clearly suggest that TRIM44 has been considered to be involved in the process of tumor metastasis and invasion. Furthermore, it may shed new light on a new prognostic marker for relevant malignant tumor. Taken together, those studies make a positive contribution to investigate the molecular mechanism by which TRIM44 participates in the migration and invasion in cervical cancer.

## Conclusion

The present study suggests that enhanced TRIM44 levels may be closely associated with the pathogenesis and poor prognosis of cervical cancer. TRIM44 serve as novel therapeutic tools for tumor therapy. A much larger study would need to effectively test our conclusion, and most importantly, investigate the TRIM44 expression in any of the other histologic subtypes and clinical factors, such as HPV infection.

## Availability of data and material

The data used and analyzed during the current study are available from the corresponding author on reasonable request.

## Abbreviations

CI: confidence interval
DAB: 3,3’-diaminobenzidine
DFS: disease-free survival
FIGO: International Federation of Gynecology and Obstetrics
HCC: hepatocellular carcinoma
HPV: human papillomavirus
HR: Hazard Rate/Ratio
mTOR: Mammalian (or mechanistic) target of rapamycin
NF-kB: nuclear factor-κB
OS: overall survival
RIPA: radioimmunoprecipitation assay
RT-PCR: Reverse Transcription-Polymerase Chain Reaction
TRIM44: tripartite motif containing 44
